# Oil droplet cataracts can mimic retinal disease

**DOI:** 10.1016/j.ajoc.2022.101321

**Published:** 2022-01-22

**Authors:** Abraham Ifrah, Janet S. Sunness

**Affiliations:** a Lewis Katz School of Medicine at Temple University, 3500 N Broad Street, Philadelphia, PA, 19104, United States; b Hoover Low Vision Rehabilitation Services and Department of Ophthalmology, Greater Baltimore Medical Center, 6569 N. Charles St, PPW 504, Towson, MD, 21204, United States; c Ophthalmology, University of Maryland School of Medicine, 655 W Baltimore Street S, Baltimore, MD, 21201, United States

**Keywords:** Oil droplet cataracts, Slit lamp

## Abstract

**Purpose:**

Oil Droplet Cataracts in adults is an elusive diagnosis for ophthalmologist. It is difficult to diagnose, and patients can suffer for years with increasingly debilitating symptoms for what is a surgically curable condition. Additionally, patients often undergo difficult and costly medical testing as well as occasionally receive improper treatment. This case series goal is to highlight this condition, showing that with careful slit lamp examination and index of suspicion one is able to appropriately diagnose this condition and avoid unnecessary testing and harm to a patient's quality of life.

**Methods:**

Nine cases of this diagnostically challenging condition seen by one of the authors of this paper (JSS) are included. All were referred for electrophysiological or careful testing for unexplained visual loss, by neuroophthalmologists and/or retina specialists. Three were suspected of having a retinal dystrophy. Many had already undergone MRI and extensive evaluations.

**Results:**

All patients were women. The average age was 45.5 years old with a range of 32–52 years of age on at their initial visit. The average length of symptoms prior to the initial visit was 3.2 years with a range of 3 months–11 years and a median of 4 years. Six had uniocular oil droplet cataracts, and three had binocular involvement. At diagnosis of the affected eyes, visual acuity ranged from 20/30–1 to 20/160 with a median of 20/65 in the affected eyes. Five patients had monocular diplopia or triplopia. Four had myopic shifts. Six patients had cataract surgery with resolution of their symptoms and restoration of good visual acuity. One patient who had been prescribed a low vision telescope for her presumed retinal dystrophy recovered to 20/20- ou, and had normalization of her electroretinogram after cataract surgery.

**Conclusions:**

This case series shows the diagnostic difficulty of this condition and the years it could take before a definitive diagnosis is made. Slit lamp examination was able to successfully diagnose this condition, although sometimes the oil droplet cataract was not seen until a later visit. Oil droplet cataracts should be considered in the differential diagnosis for a patient presenting with unexplained visual loss or acute worsening visual difficulties, and may mimic a retinal dystrophy. Once diagnosed, cataract surgery can cure this condition.

**(**Abraham Ifrah, Janet S Sunness; Oil Drop Cataracts Mimicking Retinal Disease. *Invest. Ophthalmol. Vis. Sci.* 2020;61(7):3851).

## 1 Introduction

Oil droplet cataracts are an inadequately recognized cause of visual loss, often misdiagnosed as a retinal dystrophy or neuroophthalmic disorder. There have been few reports of this condition in the literature, dating from 1946.

Oil droplet cataracts remain an elusive diagnosis for the clinician. The few cases of oil droplet cataracts previously presented in the literature showed the years patients can suffer with worsening symptoms as well as the significant amount of unnecessary testing that may be performed on these patents.^1^ Some patients were accidently given LASIK surgery or enhancement due to the absence of a proper diagnosis of an oil droplet catatact.^2^

**Fig. 1:**
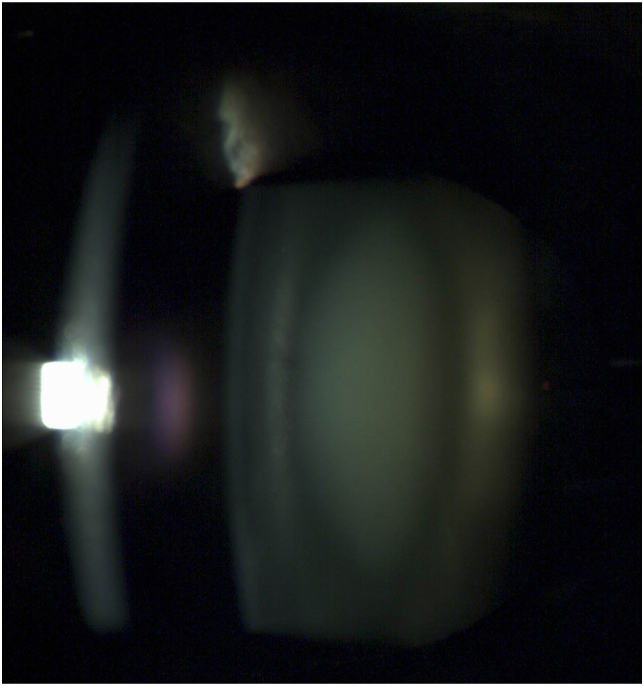
Slit lamp photograph of lens of patient 1 in Table 1. Note the lucent space between the nucleus and the cortex.

Previous researchers have appreciated the diagnostic challenges of diagnosing an oil droplet cataract^1^^,^^2^^,^.^3^ An oil droplet cataract has a small central zone separated by a clear zone from the remainder of the adult nucleus or cortex (Fig. 1). They characteristically cause blurred vision and monocular diplopia or triplopia with a myopic shift. Their appearance may be subtle, which is responsible for the delay in diagnosis or misdiagnosis. The lens changes may be more prominent on retinoscopy, when they may show a circular black region in the red reflex,or scissoring on retinoscopy.

The clinical symptoms of oil droplet cataract have been characterized as worsening visual acuity with monocular diplopia.^1^ One paper found that patients also experienced ghosting, polyopia, glare, unstable refraction, as well as losing ability to drive at night.^2^

The characterization of myopic progression, monocular diplopia with normal neuroophthalmology testing, has been well established in these patients.^1^^,^^2^^,^^3^ However, these papers have not discussed various other symptoms that may be present in these patients. The goal of this paper will be to further characterize the possible clinical features of oil droplet cataracts, most significantly, the potential for oil droplet cataracts to mimic retinal disease.

## 2 Materials and methods

Nine cases of this diagnostically challenging condition seen by one of the authors of this paper (JSS) are included. All were referred for electrophysiological or careful testing for unexplained visual loss, by neuroophthalmologists and/or retina specialists. Three were suspected of having a retinal dystrophy. Many had already undergone MRI and extensive evaluations.

## 3 Results

All patients (n = 9) were women. The average age was 45.5 years old with a range of 32–52 years of age at their initial visit. The average length of symptoms prior to the initial visit was 3.2 years with a range of 3 months–11 years and a median of 4 years. Six patients had uniocular oil droplet cataracts, and three had binocular involvement. At diagnosis of the affected eyes, visual acuity ranged from 20/30–1 to 20/160 with a median of 20/65 in the affected eyes (Table 1). Oil droplet cataracts were found to cause a wide variety of clinical symptoms (Table 2).

**Table 1:** Visual acuity at diagnosis and after surgery.

Patient	VA OD at Diagnosis	VA OS at Diagnosis	VA after Surgery
1	20/40–1+1	20/40–1+1	20/20 OU
2	20/60 + 2	20/20–1.	20/20 OD 20/25 OS
3	20/30-1	20/20	20/20–2 OU
4	20/40–2 + 2	20/20 + 2	20/20 OU
5	20/125–2+1	20/125–2+2 os	20/25 OU
6	20/70 + 2	20/160	20/20–2 OD 20/20 OS
7	20/125 + 1	20/100-2	No surgery
8	20/100	20/15-2	No surgery
9	20/50-1	20/20 + 2	No surgery

**Table 2:** Additional visual symptoms.

Clinical Finding	Number of Patients with Symptom
Diplopia or Triplopia	5
Presumed Retinal Disease	3
Myopic Shift	4
Contrast Issue	3
Glare Sensitivity	4
Light Sensitivity	6

Full-field electroretinograms were performed for 6 of the patients. Three patients had normal ERGs, and two had mildly reduced bright scotopic responses. The sixth patient had rod responses reduced to 85% of the normal amplitude, and cone amplitudes of about 65% of the normal amplitude, with slightly delayed responses.

Six patients had cataract surgery with resolution of their symptoms and restoration of good visual acuity (Table 1). One patient (the sixth described above), who had been prescribed a low vision telescope for her presumed retinal dystrophy, recovered to 20/20- ou, and had normalization of her electroretinogram after cataract surgery. Two patients were lost to follow up and 1 patient declined treatment.

## 4 Discussion

This case series shows the diagnostic difficulty of this condition and the years it could take before a definitive diagnosis is made. The condition may be more common than is reflected by the paucity of papers, and may have just been categorized as cataracts. Slit lamp examination was able to successfully diagnose this condition, although sometimes the oil droplet cataract was not seen until a later visit. We found additional clinical symptoms that were present with the diagnosis of an oil droplet cataract. Interestingly, this group of patients included only women, with a narrow age range of 32–52. The most novel presentation in this case series were the three patients that had presumed diagnosed retinal disease based on their clinical symptoms and mixed results on ERG. Thus, an oil droplet cataract should be considered in the differential if it begins to present with significant myopic progression and diplopia even in the presence of presumed retinal disease. From these nine patients, (the most to ever be covered in an oil droplet cataract case series) it would seem necessary to consider the emergence of multiple visual changes discussed here in addition to changes in visual acuity and monocular diplopia as potentially suggesting an oil droplet cataract.

## Patient consent

This retrospective study was approved by the Institutional Review Board of the Greater Baltimore Medical Center. Given the retrospective nature of this study, and the fact that all data has been deidentified, it was not necessary to obtain consent. This study disclosed no personal health information and was HIPPA compliant.

## Funding

No funding or grant support.

## Authorship

All authors attest that they meet the current ICMJE criteria for Authorship.

## Declaration of competing interest

The following authors have no financial disclosures: (AAI, JSS).

## References

[1] Heher K.L., Stark W.J., Miller N.R. (1993). Oil-drop cataracts. J Cataract Refract Surg.

[2] Soong K.H., Dastjerdi M.H. (2004). Lenticular myopia from oil-drop cataract. J Cataract Refract Surg.

[3] Hodges J.S., Marcus S., Page E. (2013). It's not a tumor, it's a cataract! Rapid myopic progression and diplopia secondary to the formation of an oil-drop cataract. Mil Med.

